# Effects of Social Media Social Comparisons and Identity Processes on Body Image Satisfaction in Late Adolescence

**DOI:** 10.5964/ejop.9885

**Published:** 2023-05-31

**Authors:** Tamara Martinac Dorčić, Sanja Smojver-Ažić, Ivana Božić, Izabela Malkoč

**Affiliations:** 1 Department of Psychology, Faculty of Humanities and Social Sciences, University of Rijeka, Rijeka, Croatia; 2 General and Vocational High School Jurja Dobrile, Pazin, Croatia; 3 Vocational-Technical High School, Split, Croatia; Trinity College Dublin, Dublin, Ireland

**Keywords:** late adolescence, body image, social comparison, identity, social media

## Abstract

One of the important developmental tasks in adolescence and emerging adulthood is the questioning of identity issues, with body image being a prominent concern. In the age of modern technology, many processes of social comparison take place on social media, which serve as an ideal platform for comparison with others. The aim of this study was to examine the effects of identity dimensions, social media use, and social media social comparison, on different domains of body image satisfaction (i.e., appearance, weight, and attribution). An online survey was conducted with 354 young people in Croatia (Mean age = 18.49, SD = 1.44; Women/girls = 78.9%). The results revealed that each of the body image domains had a different pattern of association with identity dimensions and social media social comparison. The contribution of identity dimensions was more important for evaluation attributed to others about one’s body appearance, whereas social media use and social comparison were more crucial for thoughts and feelings about appearance and weight satisfaction. Higher identity commitment and exploration were related to more positive thoughts about how others evaluate one’s appearance, regardless of social comparison. On the other hand, social media use and social media social comparison were associated with lower satisfaction with appearance and weight.

## Identity Development and Body Image During Adolescence

Identity formation is considered one of the key developmental tasks during adolescence, whereby individuals are required to question who they are, what role they play, and what they want to become (Crocetti, 2017; Erikson, 1968). Given the multitude of stresses and conflicts that modern society brings, difficulties in identity development seem to be a common problem among today’s youth (Yang et al., 2018). Compared to previous generations, individuals today face an increasing number of choices about what to value in life and how to live, in part because society has become much more complex, diverse, and interconnected (Berman et al., 2004). Therefore, the questioning of identity has extended beyond adolescence (ages 13–18) and into emerging adulthood (ages 18–29) (Arnett, 2007).

In explaining the process of identity development, several theoretical models (e.g., Balistreri et al, 1995; Crocetti, 2017) have extended the work of Erikson (1968) and Marcia (1966). Marcia (1966) proposed identity statuses based on two dimensions: exploration (i.e., actively questioning and weighing various alternatives before making a final decision) and commitment, (i.e., making identity decisions and engaging in activities to implement them). These identity dimensions are important for psychosocial functioning and adjustment. As commitment provides a sense of security and stability, it is linked to psychosocial resources and healthy adjustment such as extraversion, emotional stability, self-esteem, and well-being (e.g., Crocetti, 2017; Karaś et al., 2015). On the other hand, exploration has both beneficial and detrimental effects. It is associated with adaptive personality traits such as agreeableness, conscientiousness, openness to experience (Luyckx et al., 2012), active social cognitive strategies for evaluating self-relevant information (Crocetti et al., 2009) and well-being (Karaś et al., 2015). It is also associated with symptoms of depression and anxiety (Crocetti, 2017). This double-edged nature of exploration may be the result of coping strategies, with proactive strategies helping to build psychological resources, while avoidant coping (worrying and rumination) leads to negative outcomes (Luyckx et al., 2012).

Body image is an integral part of identity (Dittmar, 2009), and one of the most important developmental tasks during adolescence is to integrate the changing body into the person’s overall sense of self (Erikson, 1968). The identity confusion that accompanies puberty may be related to identity exploration, of which the ultimate developmental goal is the integration of personal identity (Schwartz et al., 2009). Body image is a multidimensional construct that encompasses evaluative self-perceptions, beliefs, thoughts, feelings, attitudes, and behaviors of individuals regarding their bodies (Cash & Pruzinsky, 2002). According to Mendelson et al. (2001), feelings about appearance can be distinguished from specific feelings about weight and attributional esteem. The appearance aspect of body esteem encompasses one’s general feelings about one’s appearance, whereas weight esteem specifically realtes to satisfaction with one’s weight. At the same time, attribution esteem refers to the evaluations that others make about one’s appearance and body. This distinction has also been confirmed by findings that appearance esteem, weight esteem, and attribution esteem are differently connected to different aspects of identity development (Wängqvist & Frisén, 2013).

Adolescents attach more importance to their appearance than adults and report higher levels of dissatisfaction, suggesting that appearance is a very important aspect of adolescent identity (Dittmar et al., 2000). Dissatisfaction with one’s body is associated with several consequences, including physical and mental health and low self-esteem (Davison & McCabe, 2006; Dittmar, 2009; Holland & Tiggemann, 2016). Girls and women are less satisfied with their bodies than men and worry more than men (Frederick et al., 2006; Holland & Tiggemann, 2016), which can be attributed to incorporated unattainable stereotypes about ideal appearance and weight (Mendelson et al., 2001). Pubertal changes in girls are accompanied by weight gain and accumulation of fat tissue, which distances them from the female body ideal. On the other hand, the maturation of a young man is in accordance with the male ideal based on muscularity (Davison & McCabe, 2006).

There are few studies analyzing the relationship between body image and identity (Daniels & Gillen, 2015; Nelson et al., 2018; Wängqvist & Frisén, 2013). Negative body image is usually associated with identity confusion during adolescence and emerging adulthood (Claes et al., 2014). The results confirmed the association between negative body image and greater identity distress and the positive association between greater identity commitment and more positive body esteem (Kamps & Berman, 2011; Wängqvist & Frisén, 2013). In addition, the findings suggest a possible gendered relationship between body image and identity. Using a sample of Swedish late adolescents, Wängqvist and Frisén (2013) found that young women explored identity and internalized societal body ideals more than young men, and that they had lower body esteem, while for young girls, both identity commitment and exploration were related to more positive perceptions of others’ evaluation of their appearance, for young men, stronger interpersonal identity commitment was related to more positive self-appraisals of their appearance.

## Social Media, Social Comparison, and Body Image

Because adolescents are in a sensitive phase in which they experience many drastic changes and become increasingly aware of their own physical appearance (Davison & McCabe, 2006), they are particularly sensitive to the influence of the media, which is a powerful source of sociocultural beauty ideals (Holland & Tiggemann, 2016).

For young people and emerging adults living in developed societies, digital technology and online communication have become an integral part of life in general. About 94% of 15–16 year-old students from 35 European countries reported using social media at least once per week (ESPAD, 2019). The most commonly reported time spent on social media on a typical school day averaged 2–3 hours per day, and on a typical non-school day, the average was six or more hours.

Adolescents tend to have accounts on a variety of social networks. The most commonly used social networks include WhatsApp, Instagram, Facebook and WeChat (DataReportal, 2022). Modern technologies have shifted many social relationships from private forms of interaction to public spaces, and self-presentation and self-dislosure are increasingly taking place online (Nesi et al., 2018). The internet and social media provide adolescents with new opportunities and challenges to experiment with their own identities and explore how they fit into the world around them (Noon, 2018; Spies Shapiro & Margolin, 2014; Yang et al., 2018). Peer response to online identities can play an important role in validating and strengthening self-esteem (e.g., Bergagna & Tartaglia, 2018; Meeus et al., 2019) and creating a sense of belonging and affiliation (Tobin et al., 2015).

Adolescents very often share a variety of information and photos on social media platforms, and this, combined with the power of social media for rapid and widespread communication, can have a significant impact on adolescents’ private and interpersonal worlds (Spies Shapiro & Margolin, 2014). Social media provide their users with the opportunity, and sometimes the need, to self-disclose (Spies Shapiro & Margolin, 2014). Accordingly, they provide numerous opportunities for social comparison (Cramer et al., 2016) by viewing other people's posts, photos, and personal information.

Social comparison (Festinger, 1954) is the process by which an individual evaluates their thoughts, feelings, behavior, and abilities in relation to others. It is one of many processes by which individuals gather information about their own level of physical attractiveness. A meta-analytic review (Myers & Crowther, 2009) showed that social comparison is associated with higher levels of body dissatisfaction. Specifically, unfavorable comparison leads to body dissatisfaction (Tantleff-Dunn & Gokee, 2002) and negative psycho-emotional outcomes such as depression and lower self-esteem (Noon, 2020). A systematic review of the effects of social media use on body image (Holland & Tiggemann, 2016) showed that the total time spent on social media was related to indices of body image (e.g., increased body surveillance, greater endorsement of the slimness ideal, more frequent comparison of appearance, decreased weight satisfaction, greater body dissatisfaction). Furthermore, Holland and Tiggemann (2016) detected appearance-based social comparison as one of the important underlying processes in the relationship between social media use and body image. Why do comparisons on social media tend to have negative effects? Sociocultural theory (Thompson et al., 1999) suggests that adolescents internalize the portrayal of unrealistic beauty ideals presented in media and engage in upward social comparison. Objectification theory (Fredrickson & Roberts, 1997) additionally highlights the vulnerability of women, as the female body in Western societies is viewed as an object that is primarily considered and valued based on appearance. Because this attitude is pervasive and persistent, women and girls tend to internalize it.

## Aim of the Study

Research suggests that social media are a common platform on which social comparisons occur (e.g., Spies Shapiro & Margolin, 2014), and that social comparisons are important for identity issues (e.g., Yang et al., 2018) and body image satisfaction (e.g., Jung et al., 2022; Krayer et al., 2008; Myers & Crowther, 2009; Tiggemann & Anderberg, 2020). Furthermore, distinct domains of body image (Mendelson et al., 2001) are differently connected with different aspects of identity development (Wängqvist & Frisén, 2013). Because culture can affect numerous factors that contribute to body image, further studies are needed to determine patterns of associations across national contexts (e.g., Karaś et al., 2015; Noon et al., 2021). In this study we focused on youth in Croatia, a country geographically part of Europe but characterised by transitional economies, patriarchal traditions, with gradual shift toward more individualistic values (Stojcic et al., 2020). Regarding body image, Croatian adolescent girls follow the imposed ideals of beauty widely represented by the Western media, which is also reflected in the growing desire for thinness (Rukavina & Pokrajac-Bulian, 2006; Stojcic et al., 2020). As for social media, Croatian youth use it as much as their peers aged 15–16 in other European countries (ESPAD, 2019), and this percentage is even higher in the sample consisting of 16–24 year olds (EU - 97%, Croatia - 100%; Eurostat, 2022).

Therefore, the purpose of this study was to examine the effects of identity dimensions, social media use, and social media comparison, on different domains of body image satisfaction (appearance esteem, weight esteem, and attribution esteem) among Croatian late-adolescents and emerging adults, accounting for age and gender differences.

First, we examined gender differences in social media use, social comparison, identity dimensions, and body image satisfaction. Consistent with previous literature (Bergagna & Tartaglia, 2018; Crocetti et al., 2012; Frederick et al., 2006; Holland & Tiggemann, 2016; Klimstra et al., 2010; Mendelson et al., 2001; Nesi & Prinstein, 2015; Wängqvist & Frisén, 2013), we expected to find gender differences in social media use, social comparison on social media, identity dimensions, and body image satisfaction (appearance esteem and weight esteem). Regarding attribution, we did not expect gender differences, as suggested in previous studies (Frisén et al., 2015; Nelson et al., 2018).

Second, we aimed to investigate whether identity dimensions, social media use, and social comparison, similarly predict different domains of body image satisfaction. Consistent with available evidence (Crocetti, 2017; Kamps & Berman, 2011; Nelson et al., 2018; Wängqvist & Frisén, 2013), we expected the identity dimension of commitment to be positively associated with body image satisfaction domains, whereas the identity dimension of exploration would be negatively associated with these domains. In line with previous work (Holland & Tiggemann, 2016; Myers & Crowther, 2009; Tantleff-Dunn & Gokee, 2002), social media use and social comparison on social media were expected to be negatively associated with domains of body image satisfaction.

## Method

### Participants and Procedure

The study was conducted with 354 Croatian adolescents and emerging adults aged 16 to 26 years (*M*_age_ = 18.49, *SD* = 1.44; Women/girls = 78.9%). The only inclusion requirement was for the participants to be third- and fourth-grade high school students or first-year university students.

Approval for study was obtained from the Faculty Ethics Committee. Given the unfavorable epidemiological situation caused by the COVID-19 pandemic, the research was conducted online. Prior to the survey, an invitation letter was sent to school principals. After the school principals gave consent *in loco parentis*, school psychologists received a link for the survey, which they forwarded to the students. For university students, the link was sent via internet groups and emails. Before completing the questionnaires, the participants gave their consent to participate in the study. They were informed that participation in the study was voluntary, that they had the right to opt out at any time, and that the researchers were required to keep the data confidential. It took an average of 10 minutes to complete the questionnaires. Data collection lasted from March to May 2021.

### Measures

#### The Demographic Data Questionnaire

The demographic data questionnaire included information on age (16–26 years), gender (0 = Women/girls, 1 = Men/boys), and whether the students attend high school or college (1 = high school, 2 = college).

#### The Social Media Use Questionnaire

Participants were asked if they use social media (Facebook, Instagram, Snapchat) either for posting or searching (Yes, No). If they gave an affirmative response, they were also asked to indicate how much time they spend on social media each day by choosing a response option from 1 to 6 (1 = less than 1 hour per day, 2 = 1 hour per day, 3 = 2 hours per day, 4 = 3–4 hours per day, 5 = 5–6 hours per day, 6 = 7 or more hours per day). These questions are formulated according to the measure of Use of Social Networking Sites (Sampasa-Kanyinga & Lewis, 2015). Participants who indicated that they did not use social media (*N* = 3) were excluded from further analysis.

#### The Social Comparison Scale

To measure social comparison, the Social Comparison and Feedback-Seeking Scale (MEIS-SCFS), which is part of the Motivations for Electronic Interaction Scale (MEIS; Nesi & Prinstein, 2015), was used. The scale included 10 items (e.g., *I use electronic interaction to compare the way I look with other people’s looks*). Response options ranged from 1 (does not apply to me at all) to 5 (fully applies to me). To distinguish between social comparison and feedback-seeking, a principal component analysis (PCA) with Oblimin rotation was conducted. The Cattell scree test and the Kaisser-Guttman criterion suggested two-factor solution (Factor 1–feedback-seeking, Factor 2–social comparison) that explained 72.46% of the variance. For our study, we applied only the Social Comparison subscale (with four items). The item score was summed to obtain the total score, with a higher number indicating a higher level of social comparison on social media. In the present sample, internal reliability was high (α = .88).

#### The Ego Identity Process Questionnaire

The Ego Identity Process Questionnaire (EIPQ; Balistreri et.al., 1995) is a 32-item questionnaire that assess identity exploration and identity commitment in the ideological (Occupation, Religion, Politics, Values) and personal (Family, Friendship, Dating, and Gender Roles) domains. Two statements on the commitment dimension (e.g., *I have definitely decided on the occupation I want to pursue*) and two statements on the exploration dimension (e.g., *I have questioned what kind of date is right for me*) represent each domain. Respondents indicate their level of agreement with each statement on a 6-point Likert scale from 1 (strongly disagree) to 6 (strongly agree). Scoring is reversed for negatively stated items. Item scores are summed for the total score on each dimension, with higher scores indicating higher commitment and exploration. Similar to the results of Zimmermann et al. (2010), we obtained problematic model fits for the two-factor EIPQ (χ^2^(463) = 1964.538, *p* < .001, CFI = 0.411, TLI = 0.369, RMSEA = 0.095, SRMR = 0.121), so we conducted a principal component analysis limiting Oblimin rotation to two factors. In this PCA, 12 of 16 commitment items loaded on Factor 1 (with factor loadings ranging from .36 to .63), and 10 of 16 exploration items loaded on Factor 2 (with factor loadings ranging from .33 to .69). Of the remaining 10 items (1, 2, 4, 6, 11, 13, 15, 23, 26, 30), seven items did not load on the expected factors and three showed either loading on both factors or very weak loading. These items were omitted from further analysis. The alpha coefficients for the shortened scales were .73 for commitment and .75 for exploration.

#### Body-Esteem Scale for Adolescents and Adults

The Body-Esteem Scale for Adolescents and Adults (BESAA; Mendelson et al., 2001) was used to examine body image satisfaction. The questionnaire consists of 23 items and 3 subscales. BE-Appearance (10 items) refers to general thoughts and feelings about appearance (e.g., *My appearance upsets me*). BE-Weight (8 items) examines how satisfied a person is with their weight (e.g., *My weight makes me unhappy*). BE-Attribution (5 items) refers to the person's beliefs about how the environment evaluates their body and appearance (e.g., *Other people consider me handsome*). Respondents indicate their level of agreement on a 5-point Likert scale ranging from 0 (never) to 4 (always). Negative items are scored inversely. The scales can be used together or separately and have been shown to be valid and reliable with participants over age 12. Higher total scores indicate more positive BE for a particular dimension. We tested the three-factor model proposed by authors of the original BESAA. In the Croatian sample the model had a poor fit: χ^2^(206) = 1757.581, *p* < .001, CFI = 0.719, TLI = 0.685, RMSEA = 0.145, SRMR = 0.120). Therefore, the factor structure of the scale was examined by principal component analysis with Oblimin rotation. The Kaiser-Guttman criterion and the Catell scree test suggested the extraction of three factors which explained a total of 64.13% of the variance. In the present sample, 6 of 10 appearance items loaded on Factor 1 (with factor loadings ranging from .64 to .81), 6 of 8 weight items loaded on Factor 2 (with factor loadings ranging from .62 to .92), and all 5 attribution items loaded on Factor 3 (with factor loadings ranging from .52 to.78). In addition, four appearance items (1, 6, 15, 23) and two weight items (3, 22) showed loading on more than one factor. These items were omitted from further analysis. Internal reliability for the 3 final subscales was satisfactory (BE-Attribution α = .73; BE-Appearance α = .87; BE-Weight α = .90).

## Results

The descriptives for all variables used in the study and their correlations are shown in Table 1.

**Table 1:** Descriptive Statistics and Correlations of the Study Variables

Variable	1	2	3	4	5	6	7	8	9
(1) Gender^a^	-								
(2) Age	-.08	-							
(3) Social media use	-.21***	-.17**	-						
(4) Social comparison	-.08	-.06	.29***	-					
(5) BESAA-appearance	.07	.02	-.25***	-.52***	-				
(6) BESAA-weight	.18**	-.04	-.15**	-.30***	.62***	-			
(7) BESAA-atribution	.00	.12*	-.06	.08	.21***	.25***	-		
(8) Commitment	-.02	.04	-.09	-.16**	.17**	.09	.09	-	
(9) Exploration	-.15**	.04	.09	.17**	-.15**	-.10	.25***	-.03	-
*M*	-	18.49	3.76	2.28	2.50	2.52	2.01	3.79	3.84
*SD*	-	1.44	1.06	1.10	0.99	1.14	0.83	0.73	0.83
Skewness	-	1.03	-0.36	0.74	-0.48	-0.52	0.10	0.25	-0.14
Kurtosis	-	3.84	0.25	-0.36	-0.51	-0.78	-0.24	-0.28	-0.40

^a^ 0 = women/girls, 1 = men/boys.

*p < .05. **p < .01. ***p < .001.

### Experiences With Social Media

Data on daily social media use among the surveyed adolescents and young adults showed that 99.2% of respondents used social media on a daily basis for posting or searching. The most commonly reported (40.4%) length of time spent on social media per day within the sample was 3–4 hours.

### Gender Differences in Observed Variables

To test for gender differences in social media use, social media comparison, identity dimensions, and body image satisfaction, a series of independent samples *t*-test were conducted.

Results showed that women/girls (*M* = 3.87) used social media more frequently than men/boys (*M* = 3.32), *t*(348) = 3.54, *p* = .001, Cohen’s *d* = 0.53. Women (*M* = 38.99) participated more in identity exploration than men (*M* = 35.90), *t*(351) = 2.84, *p* = .005, Cohen’s *d* = 0.38. In addition, women (*M* = 14.51) are less satisfied with their weight than men (*M* = 17.55), *t*(351) = 4.18, *p* < .001, Cohen’s *d* = 0.45. There were no significant gender differences regarding social comparison, *t*(351) = 1.48, *p* = .14, identity commitment, *t*(351) = .39, *p* = .70, BE-appearance, *t*(351) = 1.39, *p* = .17, and BE-attribution, *t*(351) = .04, *p* = .97.

### Predictors of Body Image Satisfaction

To determine whether identity dimensions, social media use, and social comparison predict different domains of body image satisfaction after controlling for age and gender, hierarchical regression analyses were conducted. We subjected each BE subscale to a separate hierarchical multiple regression analysis. Gender and age were entered in Step 1, identity dimensions in Step 2, social media use in Step 3 and social comparison in Step 4 (Table 2).

**Table 2:** Standardized Coefficients of Predictors of Body Image Satisfaction: BE-Appearance, BE-Weight, and BE-Attribution

Predictors	BE-appearance	BE-weight	BE-attribution
Step 1			
Age	.04	-.02	.11*
Gender^a^	.08	.18**	.01
*R^2^*	.01	.03**	.01
Step 2			
Age	.04	-.03	.10
Gender^a^	.07	.18**	.05
Commitment	.17**	.09	.10
Exploration	-.13*	-.06	.26***
*R^2^*	.05***	.05**	.09***
*ΔR^2^*	.04***	.01	.07***
Step 3			
Age	-.01	-.05	.09
Gender^a^	.02	.15**	.04
Commitment	.15**	.08	.09
Exploration	-.11*	-.05	.27***
SM use	-.22***	-.12*	-.06
*R^2^*	.10***	.06**	.09***
*ΔR^2^*	.05***	.01*	.00
Step 4			
Age	-.02	-.05	.09
Gender^a^	.01	.15**	.04
Commitment	.09	.05	.10*
Exploration	-.05	-.02	.25***
SM use	-.10*	-.05	-.08
Soc. comp	-.46***	-.26***	.09
*R^2^*	.28***	.12***	.10***
*ΔR^2^*	.19***	.06***	.01

*Note*. SM use = social media use, Soc. comp. = social comparison.

^a^ 0 = women/girls, 1 = men/boys.

*p < .05. **p < .01. ***p < .001.

The results showed that identity dimensions, social media use, and social comparison explained a significant proportion of the variance in body image satisfaction. The observed set of predictor variables accounted for 28% of the variance in BE-appearance, 12% of the variance in BE-weight and 10% of the variance in BE-attribution. In the final step of the hierarchical regression analyses, age did not account for variance in any of the BE measures. Gender was a significant predictor only for BE-weight (β = .15, *p* = .006). Identity commitment (β = .10, *p* = .049) and exploration (β = .25, *p* < .001) significantly predicted only BE-attribution. Identity commitment and exploration were significant predictors of BE-appearance in the previous steps, but they lost their significance when social comparison was entered. Social media use was a significant predictor only for BE-appearance (β = -.10, *p* = .039). After controlling for age, gender, and identity dimensions, social comparison negatively predicted BE-appearance (β = -.46, *p* < .001) and BE-weight (β = -.26, *p* < .001).

## Discussion

The present study aimed at investigating gender differences in social media use, social comparison, identity dimensions, and body image satisfaction. Moreover, we aimed to investigate whether identity dimensions, social media use, and social comparison, similarly predict different domains of body image satisfaction.

We found that women use social media more than men. Previous research has already found gender differences in technology use—women spend more time each day engaged in in-person communication, voice communication, non-voice phone use, Facebook and Instagram (e.g., Nesi & Prinstein, 2015). Consistent with previous research, we expected women/girls to show higher levels of social comparison on social media than men/boys (e.g., Nesi & Prinstein, 2015). However, we did not find gender differences in social comparison on social media. It is possible that the specific context of the pandemic led to greater social media orientation among young people, regardless of gender.

Similar to the results of previous research (Klimstra et al., 2010), we found that women explore their identity more than men, but the level of commitment is similar. It seems plausible that girls’ earlier physical maturation is also reflected in their earlier psychological maturation (Crocetti et al., 2012).

Contrary to our expectations, gender differences in body image satisfaction were found only for BE-weight and not for BE-appearance: women are less satisfied with their weight than men. This gender difference is consistent with previous findings (e.g., Holland & Tiggemann, 2016; Mendelson et al., 2001). Body image concerns and problematic weight control behaviors are common among adolescents and these concerns are gendered in nature, with beauty ideals differing between men and women. Thinness has been a key component of female beauty (Daniels & Gillen, 2015), whereas for men, a key component is muscularity. That may be a reason for the lack of gender difference in appearance esteem. Specifically, it seems possible that appearance is becoming increasingly important to men today as well, with a focus on a different issue—the desire to develop muscularity (Daniels & Gillen, 2015). Also, the results of previous studies have not confirmed the existence of gender differences in attribution (Frisén et al., 2015; Nelson et al, 2018).

Our results showed that identity dimensions, social media use, and social comparison were important determinants of body image satisfaction, but contrary to our expectations differently contributed to each domain of body esteem.

The identity dimensions were found to be significant for two domains of body satisfaction—appearance and attribution, but not for weight. It seems that BE-weight is a specific component of body esteem and is more related to objective parameters: Mendelson et al. (2001) found that BE-weight was the only subscale that was uniquely related to weight, with overweight individuals tending to be dissatisfied with their weight. As such, it may be less sensitive to identity processes. Consistent with available evidence (Crocetti, 2017; Kamps & Berman, 2011; Nelson et al., 2018), we found a positive association between commitment and BE-appearance. The more committed a person is to decisions related to their own identity, the more satisfied they are with their appearance. Our hypothesis about the role of exploration was only partially confirmed: exploration was negatively associated with BE-appearance but positively associated with BE-attribution. Adolescents who explore more developmental alternatives in several important identity-determining domains are less satisfied with their appearance but attribute more positive opinions about their bodies and appearance to others. This finding illustrates the double-edged nature of exploration (Luyckx et al., 2012). The positive association with attribution could be the result of exploration as a proactive strategy aimed towards building psychological resources, while the negative association with appearance could be related to worrying and rumination (Luyckx et al., 2012).

In accordance with previous work (Holland & Tiggemann, 2016; Myers & Crowther, 2009; Tantleff-Dunn & Gokee, 2002), we expected social media use and social comparison on social media to be negatively associated with different domains of body image satisfaction. Our hypothesis was only partially confirmed—social media use and social comparison are only relevant for appearance and weight satisfaction.

In terms of social media use, adolescents and emerging adults who used social media more often were less satisfied with their appearance and weight. Interestingly, in combination with social comparison, we found that social media use remained a significant predictor for BE-appearance, but not for BE-weight. Appearance seems to be the most sensitive of all the domains of body image satisfaction when using social media. Conversely, social media use and social comparison are unrelated to attribution.

Social media is known to be heavily focused on appearance, with content and messaging promoting idealized beauty standards (Jung et al., 2022). A substantial number of studies have found that greater use of social media is associated with greater body image concerns among young adults (Grabe et al., 2008), while Kim and Chock (2015) found that targeting peers’ profiles for comparison and commenting on them was related to greater body image concerns.

One of the presumed processes underlying the association between social media use and body disatisfaction is appearance-based social comparison (Holland & Tiggemann, 2016). Studies show that users compare their appearance to the idealized images they see on social media (Grabe et al., 2008). Whereas traditional media display images of celebrities and models, social media targets for social comparison of young people are largely their peers (Tiggemann & Anderberg, 2020). Social media users (e.g., Instagram users) tend to share only their best photos, carefully selected and edited (Tiggemann & Anderberg, 2020), promoting a culture of idealization. Such artificial standards for appearance represent unattainable goals for most adolescents. This process of social comparison contributes to body dissatisfaction directly or indirectly as a mediator between social media use and body image outcomes (Jung et al., 2022; Kim & Chock, 2015; Myers & Crowther, 2009). Our results support this finding: those who made more social comparisons on social media, had lower appearance and weight satisfaction. Although we may conclude that most of the impact of social media use occurs through increased use of social comparison (which in this study refers to the use of electronic interaction with the goal of comparison), part of this relationship remains unexplained. It is possible that comparison processes are activated even if we have no such intention.

### Implications

In this study, we analyzed the still insufficiently explored relationship between identity processes (e.g., exploration, commitment) and three domains of body image satisfaction, including important factors such as social media use and social media social comparison, controlling for gender and age. The study was conducted with late adolescents and emerging adults in Croatia, where this phenomenon has not yet been sufficiently researched.

In addition to the theoretical implications in relation to differentiation between body esteem domains, this study has some practical implications. For young people building their identity, social media is an important source of information that cannot be ignored. Social media offers a number of opportunities, including the possibility of social comparison, often with unattainable body ideals, which can lead to problems with body image satisfaction and identity development. If not addressed appropriately, these problems can lead to eating disorders, low self-esteem, self-confidence, etc.

Therefore, more social media literacy programs are needed to encourage critique of online content—young people should be made aware that social media users usually upload their selected and edited photos, which are likely to be far from reality. It is important to promote healthy behaviors on social media that show the real everyday life of young people, rather than unattainable ideals of beauty and lifestyle.

### Limitations and Suggestions for Future Research

The results should be interpreted with caution, keeping in mind some limitations. The relations identified in this study are correlational and it is not known, for example, whether identity dimensions cause body image satisfaction or vice versa. The sample could be more gender balanced. Only self-report measures were used, and there are some concerns which are inherent to conducting online surveys. Data for this study were collected during the COVID 19 pandemic, when most social activity occured via social media, which could affect the generalizability of the results. It is also important to consider that the adapted versions of the EIPQ and BESAA scales were used.

Future studies could analyze other variables as possible predictors of body image satisfaction (e.g., type of social media, specific reasons for social media use, active or passive type of social media use, type of social comparison, social comparison target) and use longitudinal data to examine causal relationships in more detail. A cross-cultural research into this phenomenon is needed to gain insight into possible cultural characteristics. In addition, further validation of the questionnaire measuring identity development and body satisfaction in Croatian samples is recommended.

### Conclusion

Our results suggest that each of the body image domains has a different pattern of association with identity dimensions and social media social comparison, suggesting that distinguishing between different body image domains is justified. The contribution of identity dimensions was more important for evaluation attributed to others about one’s body appearance, whereas social media use and social comparison were more crucial for thoughts and feelings about appearance and weight satisfaction. Social media use and social media social comparison were associated with lower satisfaction with appearance and weight.
